# BCMA CAR T-cell therapy in a patient with heavily pretreated relapsed/refractory POEMS Syndrome: a case report

**DOI:** 10.1007/s00277-026-07224-5

**Published:** 2026-08-08

**Authors:** Hongxiao Han, Danqing Zhao, Anan Li, Kaini Shen, Lu Zhang, Jian Li

**Affiliations:** 1 Department of Internal Medicine, Peking Union Medical College Hospital, Chinese Academy of Medical Sciences and Peking Union Medical College, Beijing, China; 2 Department of Hematology, Peking Union Medical College Hospital, Chinese Academy of Medical Sciences and Peking Union Medical College, Beijing, China; 3 Center for Rare Diseases, State Key Laboratory of Complex Severe and Rare Diseases, Chinese Academy of Medical Sciences and Peking Union Medical College, Beijing, China

**Keywords:** Relapsed/refractory POEMS syndrome, BCMA CAR T-cell Therapy, Equecabtagene autoleucel, ICATH, Hypogammaglobulinemia

## Abstract

B-cell maturation antigen (BCMA) chimeric antigen receptor T-cell (CAR T-cell) therapy is an established treatment for relapsed/refractory multiple myeloma. However, its application in POEMS syndrome remains largely unreported, despite overlapping pathogenesis involving plasma cell dyscrasia. Herein, we present a woman in her 30s with heavily pretreated relapsed/refractory POEMS syndrome who received equecabtagene autoleucel, a fully human anti-BCMA CAR T-cell product. The patient achieved a complete vascular endothelial growth factor (VEGF) response by day 14 post-infusion, with sustained remission through 12 months of follow-up. Clinical improvements included resolution of heart failure, pulmonary hypertension, and renal dysfunction, along with marked neurological recovery, enabling the patient to return to normal daily activities and occupational functioning, a status that persisted through the 16-month follow-up. Treatment-related toxicities comprised Grade 2 cytokine release syndrome and persistent hypogammaglobulinemia, without severe infectious complications. No delayed neurotoxicity or secondary malignancies were observed. This case provides evidence that BCMA CAR T-cell therapy is a safe and efficacious option for patients with relapsed/refractory POEMS syndrome.

In recent years, B-cell maturation antigen (BCMA) chimeric antigen receptor T-cell (CAR T-cell) therapy has emerged as one of the most promising approaches for plasma cell disorders, particularly in relapsed/refractory multiple myeloma (RRMM) [1–3]. The National Medical Products Administration of China has approved equecabtagene autoleucel (Eque-cel), a fully human BCMA-directed CAR-T product, for the treatment of adults with RRMM who have received at least three prior lines of therapy, including a proteasome inhibitor and an immunomodulatory agent. POEMS syndrome is a rare paraneoplastic syndrome resulting from an underlying plasma cell disorder, characterized by a collection of polyneuropathy, organomegaly, endocrinopathy, monoclonal protein, and skin changes [4]. Given the pathological similarities between POEMS syndrome and MM, BCMA CAR T-cell therapy may offer a potential therapeutic option for POEMS syndrome. Herein, we present a patient with POEMS syndrome who was successfully treated with BCMA CAR T-cell therapy, highlighting a novel and promising therapeutic approach for this challenging disease.

A woman in her 30s presented to our hospital in July 2020 with a 6-month history of bilateral lower limb numbness and pain, accompanied by gait disturbance. Physical examination revealed glomeruloid hemangiomas on the right arm and trunk. Neurological assessment using the Overall Neuropathy Limitations Scale (ONLS) demonstrated significant impairment, with scores of 4 in the upper limbs and 5 in the lower limbs. Laboratory findings were consistent with POEMS syndrome: vascular endothelial growth factor (VEGF) was markedly elevated at 5106 pg/mL; however, serum immunofixation electrophoresis and serum protein electrophoresis were negative, possibly due to the limited sensitivity of these assays in detecting low-level M protein [5]. Serum free light chain (sFLC) analysis revealed a lambda-restricted pattern: sFLC-lambda was 83.0 mg/L (reference range, 5.7–26.3), sFLC-kappa 22.2 mg/L (reference range, 3.3–19.6), with a kappa/lambda ratio of 0.267. Additional notable findings included elevated β-crosslaps at 1.52 ng/mL and thrombocytosis (platelet count 514 × 10⁹/L). Initial imaging included a CT scan revealing multiple enlarged retroperitoneal lymph nodes and an MRI showing sclerotic bone disease at S1. A subsequent core needle biopsy of the S1 sclerotic lesion confirmed the presence of a plasmacytoma. A diagnosis of POEMS syndrome was established according to the diagnostic criteria [4]. The patient’s treatment course is summarized in Fig. 1A. In August 2020, she initially received three cycles of lenalidomide and dexamethasone (Rd), but achieved only stable disease. This was followed by autologous stem cell transplantation (ASCT) in November 2020, which induced a partial response lasting 35 months (Fig. 1B). Upon disease progression in October 2023, she received multiple subsequent lines of therapy, including bortezomib plus dexamethasone (BD), bortezomib, pomalidomide, and dexamethasone (VPD), daratumumab, carfilzomib, and dexamethasone (DKD), and daratumumab, pomalidomide, and dexamethasone (DPD). However, all these regimens proved ineffective, as evidenced by rising VEGF levels (Fig. 1B), recurrent lambda-restricted sFLC abnormalities (sFLC lambda 96.6 mg/L; kappa/lambda ratio 0.083), and the development of complications including heart failure, pulmonary hypertension, renal insufficiency, and polyserositis. Given the patient’s refractoriness to multiple regimens, BCMA CAR T-cell therapy was administered. Lymphodepleting chemotherapy consisting of fludarabine (20 mg/m²) and cyclophosphamide (300 mg/m²) was given on days − 5, −4, and − 3 prior to CAR-T cell infusion. On day 0, the patient received a single infusion of anti-BCMA CAR T-cells at a total dose of 1 × 10⁶ cells/kg. The patient experienced Grade 2 cytokine release syndrome (CRS) on day + 2, graded according to established criteria [6]. Clinical manifestations included fever (peak temperature 38.1 °C) and elevated acute-phase reactants (hsCRP 15.6 mg/L, from a previously normal baseline), with no microbiological evidence of infection. A single dose of tocilizumab (8 mg/kg) was administered for anti-inflammatory management, resulting in complete resolution of fever on day + 5. The patient developed Grade 2 early immune effector cell-associated hematotoxicity (ICAHT) following infusion [7], which was managed with supportive care including granulocyte colony-stimulating factor, erythropoietin, and red blood cell transfusions. At the 8-month post-infusion assessment, the patient’s Grade 3 late ICAHT (asymptomatic cytopenia) had resolved and remained normal thereafter (Fig. 1C). Hypogammaglobulinemia (IgG < 4 g/L) developed on day + 6, prompting intravenous immunoglobulin (IVIG) replacement (20 g daily for 3 days) during the initial three months. Throughout the 12-month follow-up, hypogammaglobulinemia persisted; however, the patient experienced no severe recurrent infections. Notably, the patient did not develop immune effector cell-associated neurotoxicity syndrome.

**Fig. 1:**
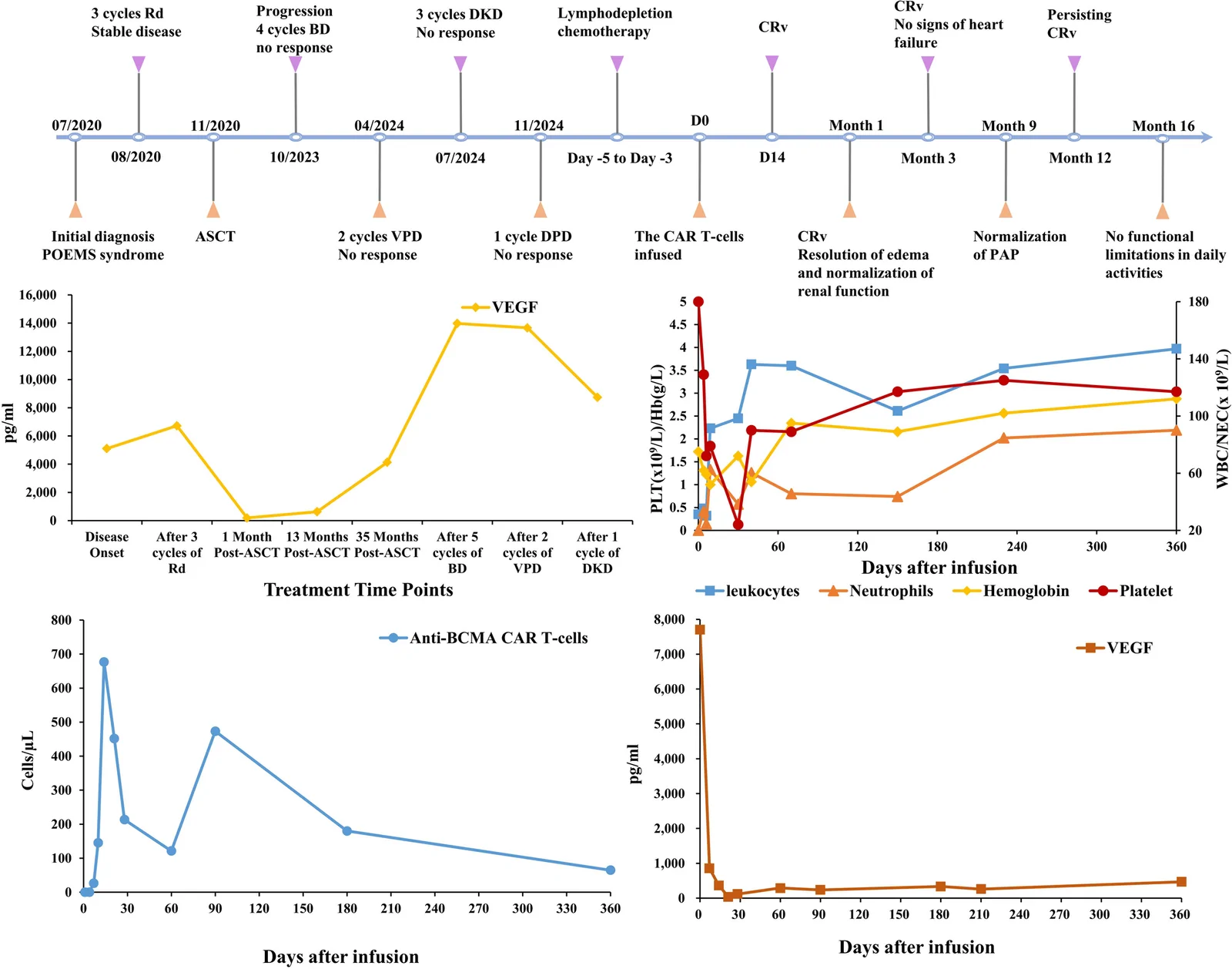
Patient's treatment timeline and response. (**A**) Overview of the patient's clinical management throughout the disease course. (**B**) Serial serum VEGF levels measured by ELISA during sequential lines of therapy, demonstrating no response. (**C**) Changes in complete blood count over the 1-year follow-up period. (**D**) Quantification of anti-BCMA CAR-T cell engraftment by flow cytometry (cells/µL). (**E**) Serial serum VEGF levels measured by ELISA over 1 year after CAR-T infusion, demonstrating a marked decrease

In the following months, longitudinal monitoring demonstrated that CAR-T expansion peaked at day + 14, with persistence detectable for up to 12 months post-infusion (Fig. 1D). The patient achieved a complete VEGF response (CRv) [8] as early as day + 14, with sFLC levels also normalizing at this time point. Both responses were sustained through the 12-month follow-up (Fig. 1E). At the 1-month follow-up, the patient’s creatinine level had normalized, accompanied by resolution of peripheral edema. Subsequent evaluations revealed sustained clinical improvement. By 3 months post-treatment, the patient exhibited no signs of heart failure, had returned to work, and demonstrated ONLS scores of 0 in the upper limbs and 2 in the lower limbs. Pulmonary artery pressure normalized by the 10-month assessment. At the 12‑month assessment, the patient remained in CRv (Fig. 1E). At the subsequent 16‑month follow‑up, she continued to have no functional limitations in daily activities.

To our knowledge, this case represents the first successful application of commercially available Eque-cel for POEMS syndrome in China. The patient, who was refractory to multiple prior lines of therapy, including immunomodulatory drugs, proteasome inhibitors, anti-CD38 monoclonal antibody, and ASCT, achieved a deep and sustained disease remission, highlighting the potent efficacy and safety of this approach in POEMS syndrome.

Previous studies have validated the efficacy of BCMA CAR T-cell therapy in RRMM [1–3]. In the FUMANBA-1 trial [9], which enrolled 103 patients with RRMM treated with eque-cel, the overall response rate was 96.0%, with 74.3% of patients achieving CR or better. The 12-month progression-free survival (PFS) rate was 78.8%, and the median PFS was not reached. The rationale for employing BCMA CAR T-cell therapy in POEMS syndrome stems from its shared pathophysiological origin with MM. In this case, VEGF normalized rapidly by day + 14 post-infusion and then remained at a sustained CRv. This early effect is unlikely to be attributable to lymphodepleting chemotherapy, but rather reflects the potent anti-plasma cell activity of BCMA CAR T-cells. The concurrent recovery of cardiac, renal, and neurological function further corroborates the clinical benefit of this approach in POEMS syndrome.

The first reported case of BCMA CAR-T therapy in POEMS syndrome used a non-standard CAR-T product in a patient who had received only the Rd regimen prior to treatment [10]. Recently, Williams et al. [11] described a relapsed POEMS case treated with commercial ciltacabtagene autoleucel with predominant neuropathy and a 6-month follow-up. By contrast, our patient presented with multiorgan involvement but developed only Grade 2 CRS, a lower inflammatory reaction, and maintained a sustained response for 16 months, exceeding the earlier follow-up duration. The fully human CAR T construct used here, with its lower immunogenicity and enhanced persistence, likely contributed to this outcome.

The patient developed Grade 2 CRS in the early post-infusion period, which resolved rapidly. However, the patient experienced long-term CAR T cell-associated toxicities, including ICAHT and hypogammaglobulinemia, the latter persisting through the last follow-up. In a phase I trial of fully-human BCMA CAR T-cell therapy for MM [12], late ICAHT was reported in 80% of patients (29/36), with Grade 3 in 28% (8/29) and Grade 4 in 3% (1/29). In another phase I trial of idecabtagene vicleucel for RRMM [1], patients with persistent Grade 3/4 cytopenias at 1 month recovered to Grade ≤ 2 in a median of 1.9 months (neutropenia, *n* = 52) and 2.1 months (thrombocytopenia, *n* = 62), respectively. In the present case, ICAHT recovery was delayed until 8 months, potentially linked to the presence of neutropenia and anemia before CAR-T cell administration and a history of multiple prior lines of therapy. Compared with patients receiving CD19 CAR T-cell therapy, those treated with BCMA-targeted CAR-T cells experience more profound humoral immune impairment, likely due to the depletion of CD19-negative, BCMA-positive long-lived plasma cells, which leads to a higher incidence of prolonged hypogammaglobulinemia [13]. Within 60–90 days after BCMA CAR-T infusion, hypogammaglobulinemia occurs in 69% of patients, and persists for over one year in 42%-47% of cases [13, 14]. Consistent with previous reports [14–16], the patient did not develop severe infectious complications despite experiencing prolonged ICAHT and hypogammaglobulinemia. Currently, randomized clinical trial evidence supporting prophylactic IVIG replacement following CAR-T therapy is lacking. Although long-term hypogammaglobulinemia increases the risk of infections, severe infections after CAR T-cell therapy most frequently occur within the first month post-infusion [15, 16]. After the initial three months of IVIG replacement therapy, the patient received no further immunoglobulin despite persistent hypogammaglobulinemia, and no severe infections were documented during follow-up.

In conclusion, this case confirms the safety and efficacy of BCMA CAR T-cell therapy in a patient with relapsed/refractory POEMS syndrome, providing key preliminary evidence for its application in this population. While further experience and long-term follow-up are needed to optimize its use, the present findings strongly support BCMA CAR T-cell therapy as a promising and viable therapeutic strategy for patients with relapsed/refractory POEMS syndrome.

## Data Availability

No datasets were generated or analysed during the current study.
